# Full Breathing

**Published:** 1884-01

**Authors:** 


### HALL'S JOURNAL OF HEALTH AND MISCELLANY.

69

MONTHLY.

E. H. GIBBS, A. M., M. D., EDITOR.

FOUNDED IN 1854.

### FULL BREATHING.

WE have always maintained that the most important agent for the preservation of health and the cure of disease is a full and constant supply of pure air for the lungs.

All the plans for systematizing exercise by combining it with proper pleasures should be encouraged. Horseback riding, walking, bicycling, foot racing and athletic sports are hardly less valuable for the increased quantity of air that they compel us to consume than from the exhilarating and healthful effects produced in our minds.

In order to be of real value, exercise should be regular and, if possible, in the open air, and sufficient each day for the wants of the system; but never excessive. Extremes are always dangerous. Gymnasiums have caused the breaking down of hundreds of young men.

Fearing to go out in inclement weather is a vain fear. There may be a day occasionally during the Winter when it might be prudent to remain indoors, but with proper attention immediately afterward no well person while exercising is likely to be the worse for a thorough drenching or wet feet.

Air is the best of all blood purifiers, and the more thoroughly the lungs do their work, the purer the blood, and the less liability will there be to disease.

I believe that the chief cause of pneumonia is breathing impure air, and next to this over-eating. Both tend to befoul the blood. The victims of this disease are principally among "good livers," who do not take sufficient exercise in the open air. The disease seldom attacks those whose occupations require them to spend much of their time out of doors.

The main object of exercise, then, is to compel the breathing of as much air as is required for the elimination of effete matters from the blood, and the reward is an almost certain immunity from all forms of disease.

